# Perceptions of Cannabis Use and Its Benefits and Risks Among Breastfeeding Mothers

**DOI:** 10.1089/whr.2024.0021

**Published:** 2024-05-06

**Authors:** Zane Boerner, Cristina Natha, Teresa Baker, Christine D. Garner

**Affiliations:** ^1^ School of Medicine, Texas Tech University Health Sciences Center, Amarillo, Texas, USA.; ^2^ Department of Internal Medicine, University of Texas Health Science Center, Houston, Texas, USA.; ^3^ Department of Obstetrics and Gynecology, Texas Tech Health Sciences Center, Lubbock, Texas, USA.; ^4^ InfantRisk Center, Texas Tech University Health Sciences Center, Amarillo, Texas, USA.

**Keywords:** cannabis, marijuana, medical marijuana, breastfeeding, lactation

## Abstract

**Background:**

Approximately 5% of breastfeeding women report using cannabis. Little is understood about perceived benefits and risks of cannabis use; thus, this study aimed to fill this gap.

**Methods:**

An anonymous online survey was conducted from 2018 to 2019 among breastfeeding women (*n* = 1516) who used cannabis. Data collected included demographics, frequency and timing of cannabis use, perceived effects in infants, and repercussions experienced. Analyses included descriptive statistics; chi-square and *t*-tests were used to test differences between groups (SPSSv28). A subset (*n* = 413) left open-text responses about cannabis and its perceived risks and benefits. Content analysis and ATLAS.ti were used for open-ended responses.

**Results:**

Two-thirds (67%) of participants were “not at all” concerned that cannabis use while breastfeeding affected their baby. Only 3% attributed symptoms in their infants to cannabis use; symptoms were perceived as positive or negative. Interestingly, 45% (*n* = 603) altered timing of cannabis use relative to breastfeeding to avoid exposing their infant to cannabis. Most mothers (85.8%) reported no changes in their breast milk supply. Few respondents were investigated by Child Protective Services (6.9%) or arrested (3.8%) for cannabis use. In open-ended responses, three themes emerged about the perceptions of cannabis use while breastfeeding: (1) cannabis preferred to address medical concerns, (2) positive impact of cannabis on quality of life for mothers and their children, and (3) concerns about negative consequences.

**Conclusion:**

Breastfeeding mothers who used cannabis reported positive perceptions of cannabis as a safer alternative to medications, yet concerns existed about legal repercussions. Understanding maternal perceptions may be useful in developing successful approaches to counseling mothers about cannabis use.

## Introduction

Medical cannabis is legal in 38 U.S. states, 3 territories, and the District of Columbia, and nonmedical cannabis is legal in 25 states, 2 territories, and the District of Columbia.^1^ As more states legalize cannabis, its use has become more accepted. A study in 2020 showed women living in states with legalized recreational cannabis were 2.2 and 1.7 times more likely to use cannabis prenatally and postpartum, respectively, compared with women in states where recreational cannabis remained illegal.^2^ Simultaneously, concentrations of Δ9-tetrahydrocannabinol (THC), the psychoactive component in cannabis, have risen from 3% in 1980 to 14% in 2019.^3^

THC can cross the placenta and cross into breast milk resulting in infant exposure.^4,5^ Current evidence suggests an association between cannabis use during pregnancy and fetal growth restriction, preterm birth, stillbirth, and increased neonatal intensive care unit admission.^6^ Prenatal exposure may also increase the risk of long-term neurobehavioral effects such as decreased reasoning and attention.^7^ However, evidence of adverse development due to cannabis exposure is mixed and difficult to study because of confounding variables such as differences in dose and duration of exposure.^6^ Owing to limited data evaluating the effects of cannabis on infants during breastfeeding, the American College of Obstetricians and Gynecologists (ACOG) discourages cannabis use while breastfeeding, and the Academy of Breastfeeding Medicine recommends counseling mothers to reduce or eliminate use of marijuana.^8,9^ There is currently no standard recommendation for cannabis use and timing breastfeeding.

An estimated 5% of pregnant and breastfeeding women report using cannabis.^10,11^ In postpartum individuals and women who used cannabis during either pregnancy or breastfeeding, perinatal cannabis use has been reported to address mental health problems (anxiety and stress), conditions related to pregnancy (nausea and vomiting), and pain.^12–14^ A study that analyzed anonymous data from HealthTap, an online digital platform that provides free access to online health information, and a study of Jamaican women who used cannabis while pregnant reported more general benefits including improved general health, spiritual well-being, and improved mood.^14,15^ Only half (46%–54%) of women who used cannabis during pregnancy reported using it to get high.^12,13^

Perceived safety of cannabis is complex and influenced by many factors including opinions of health care providers, information read online, personal experiences, and anecdotal information.^16^ A 2020 study reported that around 20% of women perceived no risk with weekly cannabis use; lower perceived risk has been associated with higher cannabis use during pregnancy.^17,18^

Despite reports of some perceived safety during pregnancy, 92% of postpartum individuals completing the Pregnancy Risk Assessment Monitoring System (PRAMS) questionnaire across seven states believed that cannabis use while breastfeeding was unsafe.^11^ Those same women were less likely to breastfeed and had shorter duration of breastfeeding compared with women who believed it was safe.^11^ This study aimed to understand mothers’ perceptions of risks and benefits of cannabis use while breastfeeding.

## Material and Methods

### Survey development

An online cross-sectional survey was developed by researchers at the InfantRisk Center, Texas Tech University Health Sciences Center to be conducted among mothers who used cannabis while pregnant or lactating. The survey was pretested and revised based on feedback from mothers who had used cannabis.^12^ The survey contained 88 questions in 8 sections and has been published.^19^ Demographic data collected included mother’s age, age of first cannabis use, race, income, age of youngest child, racial/ethnic group, marital status, highest level of education, and country of residence.

Specific questions were asked about cannabis use to assess the mother’s cannabis-related behaviors, perception of cannabis and its effects, and perceived consequences of cannabis use. Cannabis-related behaviors included smoking to get high (yes/no), frequency of use while breastfeeding (frequency in a week and frequency in a day), and if they intentionally altered their cannabis use to avoid exposing their infant (breastfed before using cannabis; waited longer after using cannabis to breastfeed; I did not alter my use of cannabis relative to breastfeeding).

Questions about perceived effects of cannabis included: if they considered it a more natural alternative to medications (yes/no), concern about it affecting their baby (not at all concerned to very concerned), if they noticed symptoms in their baby while breastfeeding (yes/no and free response), and if there was a noticed change in breast milk supply (increase, no change, decrease). Mothers who reported observed symptoms in their baby were provided an opportunity to write in the symptom; these responses were split into five categories (sleepy, calm, advanced, delayed, and other negative symptoms).

Three questions pertained to legal consequences of cannabis use including being arrested for cannabis, being investigated by Child Protective Services (CPS), and having a child removed by CPS.

The last question of the survey was an open-text question to which all survey participants were provided an opportunity to respond: “Is there anything you would like to tell us? Feel free to add anything you would like to say about this survey or any topic you’d like for us to ask more about.”

### Survey distribution

The survey was distributed online through social media pages of the InfantRisk Center (which has broad national following) and the study investigators and was also distributed to a support group for mothers who used cannabis during pregnancy or while breastfeeding. The InfantRisk Center website hosted the survey and made it available to anyone who viewed the website. This study used a combination of convenience and snowball sampling.

The Qualtrics (Qualtrics, Provo, UT, USA) platform was used and was set up to limit submissions to prevent multiple participation in this survey. The survey was completed anonymously. Respondents were not compensated for completing the survey. Data were collected from May 2018 to April 2019.

### Study sample

**FIG. 1.**
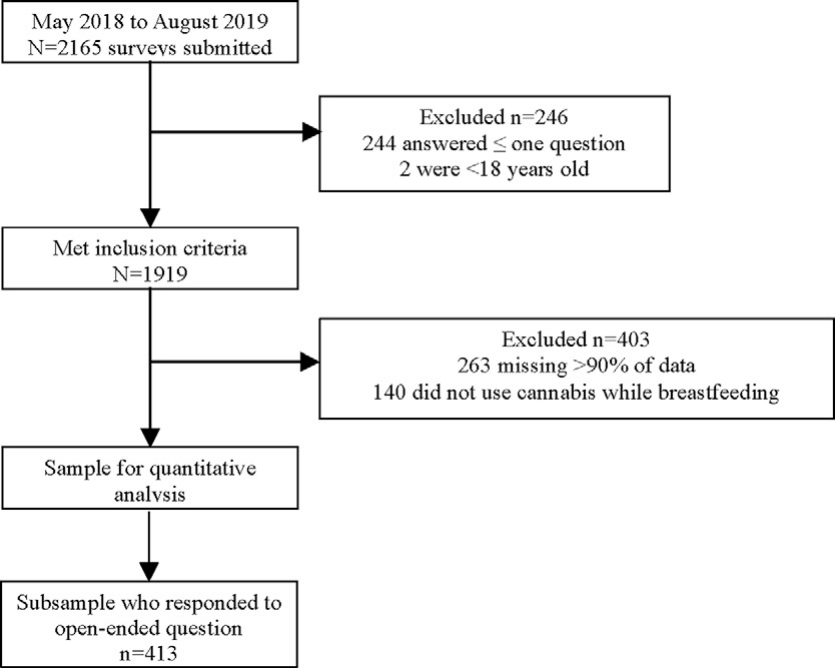
Study sample flow chart.

This study includes data from mothers who used cannabis while breastfeeding (*n* = 1516). Of 2165 survey responses, 1919 met inclusion criteria (244 were excluded for answering one or fewer questions and 2 were excluded for being under 18 years of age). Another 263 were missing over 90% of the data; 140 were excluded for not using cannabis while breastfeeding, leaving 1516 responses used in the quantitative analysis. A subsample of 413 responded to the open-ended question and were used for the qualitative portion of the study (Fig. 1). Completion of this survey was considered consent to participate. This study was approved by the Institutional Review Board at Texas Tech University Health Sciences Center (A18-4037).

### Data analysis

Data were analyzed using IBM SPSS Statistics for Windows, v. 29 (IBM Corp., Armonk, NY, USA), descriptive statistics are reported, and chi-square tests for categorical data and *t*-tests for continuous data are reported for differences between groups. Open-ended responses were analyzed through qualitative content analysis and ATLAS.ti Mac v. 9. Three researchers read all comments and identified concepts that occurred in the data. The team developed a list of codes and discussed the concepts and coding at weekly meetings. The data were coded in three rounds. Multiple codes could be added to a single quote, and coding was agreed upon during regular meetings. Once coded, recurrent themes were identified, and the codes were grouped accordingly.

Comparative statistics between respondents included in the quantitative and qualitative study are reported.

## Results

### Respondent characteristics

Among the 1516 respondents included in this analysis, 91% lived in the United States, 6.4% lived in Canada, and 3.1% lived in other countries (Table 1). The mean maternal age was 30 years and mean parity was 2. Just over half (56.1%) of the mothers had given birth 12 months or more prior to responding to the survey. Nearly two-thirds of survey respondents were married (64%), and a majority had some post-high school education (80.7%). A majority (78.2%) of respondents were White non-Hispanic, 8% were Hispanic, and 14% were Black, indigenous, or other people of color. Respondents were distributed across four income categories evenly.

**Table 1.** Characteristics of Survey Respondents Who Used Cannabis While Breastfeeding

		Responded to open-ended question			
Characteristics	Total	Y	N	Test statistic^a^	*p*-Value
Maternal age, mean (SD)	29.98 (6.1)	30.38 (5.8)	29.83 (6.3)	−1.042	0.15
Parity, mean (SD)	2.018 (1.1)	1.973 (1.1)	2.035 (1.1)	−0.951	0.17
Age of first cannabis use, mean (SD)	16.78 (4.1)	16.78 (4.4)	16.78 (4.0)	0.022^b^	0.491
Youngest child’s age *n* (%)				1.972	0.373
Months	365 (24.2)	99 (24.1)	266 (24.2)		
6 to <12 months	298 (19.7)	72 (17.5)	226 (20.6)		
12 months or older	846 (56.1)	240 (58.4)	606 (55.2)		
Marital status, *n* (%)				6.125	**0.047**
Married	939 (63.7)	249 (61)	690 (64.8)		
Single	311 (21.1)	82 (20.1)	229 (21.5)		
Other	223 (15.1)	77 (18.9)	146 (13.7)		
Race/Ethnicity, *n* (%)				0.298	0.862
White Non-Hispanic	1028 (78.2)	274 (77.2)	754 (78.5)		
Hispanic	108 (8.2)	30 (8.5)	78 (8.1)		
BIPOC	179 (13.6)	51 (14.4)	128 (13.3)		
Education level attained, *n* (%)				12.737	**0.002**
High school or less	287 (19.2)	61 (14.8)	226 (20.9)		
Some post-high school	780 (52.3)	210 (51)	570 (52.8)		
Bachelor’s or higher	424 (28.4)	141 (34.2)	283 (26.2)		
Income, *n* (%)				0.285	0.963
25K or less	261 (20.4)	70 (19.8)	191 (20.6)		
26K–50K	404 (31.5)	109 (30.9)	295 (31.8)		
51K–75K	290 (22.6)	82 (23.2)	208 (22.4)		
76K or more	326 (25.4)	92 (26.1)	234 (25.2)		
Country, *n* (%)				1.354	0.51
United States	1336 (90.6)	357 (89.7)	979 (90.9)		
Canada	94 (6.4)	30 (7.5)	64 (5.9)		
Other	45 (3.1)	11 (2.8)	34 (3.2)		
Smokes tobacco, *n* (%)	206 (15.3)	45 (10.9)	161 (17.3)	8.84	**0.003**
Drinks alcohol, *n* (%)	606 (45.2)	177 (42.9)	429 (46.2)	1.274	0.259

Bolded *p*-value indicates statistical significance at a *p* < 0.05.

^a^ *t*-test was used for continuous variables; chi-square was used for categorical variables.

^b^ Did not meet criteria for equal variance by Levene’s test.

SD, standard deviation; BIPOC,  Black, indigenous, or other  people of color.

**Table 2.** Responses to Survey Questions Related to Cannabis Use

		Responded to open-ended question			
	Total	Yes	No	Test statistic	*p*-Value
Used cannabis while pregnant, *n* (%)	1173 (77.4)	339 (82.1)	834 (75.6)	7.186	0.007
Used cannabis to get high, *n* (%)	754 (53.4)	226 (55.1)	528 (52.7)	0.659	0.417
Cannabis use frequency while breastfeeding, *n* (%)				0.559	0.455
Less than daily	686 (51.7)	199 (50.1)	487 (52.4)		
Daily user while breastfeeding	641 (48.3)	198 (49.9)	443 (48.3)		
Investigated by CPS for cannabis use, *n* (%)	93 (6.9)	35 (8.5)	58 (6.2)	2.306	0.129
Been arrested for using cannabis, *n* (%)	51 (3.8)	17 (4.1)	34 (3.6)	0.179	0.672
Noticed change in milk supply while using cannabis, *n* (%)				0.067	0.967
Increased my milk supply	167 (12.3)	51 (12.5)	116 (12.3)		
No change in my milk supply	1163 (85.8)	351 (85.8)	812 (85.8)		
Decreased my milk supply	25 (1.8)	7 (1.7)	18 (1.9)		
Altered timing of cannabis use to avoid exposing infant, *n* (%)				2.933	0.231
Yes, I breastfed before using cannabis	484 (35.7)	143 (34.8)	341 (36.2)		
Yes, I waited longer after using cannabis to breastfeed	119 (8.8)	90 (9.5)	29 (7.1)		
No, I did not alter my use of cannabis relative to breastfeeding	751 (55.5)	512 (54.3)	239 (58.2)		
Concerned cannabis is affecting your baby, *n* (%)				0.536	0.970
1—Not at all concerned	804 (67.2)	252 (67.7)	552 (67.0)		
2	279 (23.3)	84 (22.6)	195 (23.7)		
3	51 (4.3)	17 (4.6)	34 (4.1)		
4	42 (3.5)	12 (3.2)	30 (3.6)		
5—Very concerned	20 (1.7)	7 (1.9)	13 (1.6)		
Noticed symptoms in baby related to cannabis use while breastfeeding, *n* (%)	40 (2.6)				

Test statistic for continuous variables is a *t*-test, and for categorical variables is a chi-square.

CPS, Child Protective Services.

The average age of first cannabis use was 17 years. Most mothers (77.4%) also used cannabis while pregnant (Table 2). A slim majority (52%) used cannabis less than daily while breastfeeding, and 53% of the respondents reported using cannabis to get high; 55% reported drinking alcohol while 15% reported smoking tobacco.

### Results of closed-ended survey questions

Two-thirds (67.2%) of respondents reported being “not at all concerned” about cannabis use while breastfeeding affecting their baby, whereas 1.3% reported being “very concerned” (Table 2). Nearly all (98%) believed that cannabis was a safer alternative to medication.

Only 2.9% (*n* = 40) believed their babies displayed symptoms from use of cannabis while breastfeeding. The symptoms reported in written-in responses were organized into five categories, some were perceived as positive effects and others as negative effects. Sleepiness was reported the most (*n* = 11); some mothers perceived it as a positive symptom: “sleeps well at night”; others described it neutrally: “tiredness.” Some mothers believed their babies were more advanced (*n* = 5) or calm (*n* = 6) because of their cannabis use. Others reported verbal/social delays (*n* = 3) or other negative consequences including irritability or failure to thrive (*n* = 8) due to cannabis use while breastfeeding.

Nearly half (44.5%) of respondents intentionally altered the timing of cannabis use relative to breastfeeding. Breastfeeding before cannabis use was reported by 35.7% of respondents and waiting longer after cannabis use to breastfeed was reported by 8.8%. Interestingly, altering the timing of cannabis use was not associated with perceived symptoms in the infant (chi-square 0.15, *p* = 0.7), possibly because of few mothers reporting perceived symptoms. Most respondents (85.8%) also did not report changes in milk supply while using cannabis; 12.3% of mothers reported an increase and 1.8% reported a decrease in their milk supply.

Most respondents had not encountered legal repercussions related to cannabis use; 6.9% reported being investigated by CPS and 3.8% reported having been arrested for cannabis use.

### Comparison of open-ended question respondents to nonrespondents

Respondents to the open-ended question were similar to the nonrespondents. They differed on marital status, education, use of cannabis during pregnancy, and tobacco smoking. The mothers who provided open-text responses more often chose “Other” for marital status (*p* = 0.047), were more educated (*p* = 0.002), more often used cannabis while pregnant (*p* = 0.007), and were less likely to smoke tobacco (*p* = 0.003).

### Qualitative open-ended question results

Open-text responses (*n* = 413) expanded on results from the closed-ended questions. They revealed new concepts that had not been addressed in the closed-ended survey questions. Three themes emerged (example quotations in Table 3). The first theme related to cannabis being preferred over medications. Theme two centered on the perceived positive impact of cannabis on quality of life for parents and their children. The third theme described concerns from respondents about possible consequences related to their perinatal cannabis use.

**Table 3.** Themes and Subthemes with Example Quotations from Open-Ended Responses Provided by 413 Respondents

Themes and subthemes	No. of responses^a^	Example quotations
Cannabis preferred to address medical concerns (*n* = 202)		
“Saved” from medical conditions or symptoms	195	1) “Please understand, MJ saved my children and as sick as I was, I could have lost them, or even my own life, without the healing properties of this herb.” (ID #1505) 2) “I smoked with both of my kids to help aid with hyperemesis gravidarum. Marijuana literally saved my life when prescribed drugs did nothing.” (ID #1321)
Fewer or better side effects than prescription medications	52	3) “I was prescribed various medications over the course of about 3 years including lithium, abilify, and trazodone. I was able to completely stop taking prescription medications, which had terrible side effects, with use of cannabis.” (ID #1140) 4) “Other drugs give me so many side effects and cannabis has none.” (ID #1841) 5) “I was put on an antidepressant that [my daughter] reacted horribly to so cannabis seemed like a safer choice with less side effects.” (ID # 2051)
Cannabis perceived as more natural and safer	34	6) “Consuming while pregnant never worried. This is an ancestral herb used for thousands of years.” (ID #933) 7) “My reasoning for using marijuana during breastfeeding was that if doctors were prescribing Zoloft and other antidepressants and saying those were okay to use, how could marijuana be any worse as a natural substance.” (ID #1281) 8) “I have many medical issues, and the prescriptions I take for them my Drs warned could affect my baby. Cannabis is natural and to me safer.” (ID #1683)
Shared decision-making	27	9) “After talking to multiple doctors, OBs, midwives, my pharmacist, and high risk OBs, everyone felt that it would likely be safest for my baby and for me to continue using my low dose of cannabis and having my chronic conditions controlled vs stopping it and not having my conditions well controlled.” (ID #76) 10) “Having been on several medications myself that did not work or had unwanted side effects, I knew I had to do something. We made the decision as a family.” (ID #14) 11) “I did research in Cannabis use during pregnancy and breastfeeding, I spoke to other Moms.” (ID #484)
Positive impact on parent and child (*n* = 77)		
Being a better parent	28	12) “I use it to function. If I don’t have it, I am angry and irritable. I have extreme anxiety and I can’t leave my house. Consuming this plant helps me function as a better parent and partner. (ID #1105) 13) “Cannabis helps me maintain a positive and multifaceted perspective and I think it helps me be a more patient and engaged parent.” (ID #2099)
Children exposed perceived to exceed milestones	54	14) “I have smoked cannabis my entire pregnancy and during breastfeeding still going strong and my son is very advanced in everything … With my first I did not smoke during pregnancy, and I did not breastfeed. He was extremely difficult as a newborn … but I've noticed that the son I did smoke with vs the one I didn't; the one I did is much more advanced in a lot of areas. (ID#1276) 15) “My oldest child weighed 6lbs at birth, my 2nd child 6lbs 5 oz. Birth weight & cognitive development were stressed in the warnings I received when marijuana & pregnancy came up in conversation. My 2nd child surpassed their older sibling in hitting first year milestones.” (ID #1284)
Children were more expressive and happier	18	16) “From day one she has been jubilant and alert, she is ‘talkative’ and incredibly expressive, she learns quickly and adapts well to change and new environments.” (ID #1121) 17) “I used cannabis w/ my first preg but not with my second as I moved to a state where it then became illegal … Understanding of the fact that all children are different and unaware if it’s related to the cannabis, my first child was much easier to console, had no issues with feeding and slept longer periods of time. Second child has difficulties sleeping, eats like a pig and is hard to console.” (ID # 1895)
Concerns about potential negative consequences (*n* = 42)		
Timing breastfeeding before cannabis use	26	18) “When I do medicate, it's after my child breastfeeds and he does not sleep in the same bed with me if I've used cannabis. We usually co-sleep, but on those occasions, he sleeps either in the swing or mini-crib in my room.” (ID # 1756) 19) “I used it only in the evenings after my son went to bed and would not breastfeed for at least 4 hours (we gave a bottle of my breast milk pumped earlier in the day).” (ID #1674)
Avoiding smoke exposure	13	20) “After using cannabis, I brush my teeth, wash my face, hands, and arms, and change my clothes to reduce smoke exposure to my child.” (ID #844) 21) “I only vaped or ate cannabis while pregnant because I felt the most dangerous part was the smoke itself … For me personally, while breastfeeding, I will only consume after my kids go to bed because otherwise, I feel paranoid. (ID #1218)
Possible hospital repercussions or CPS involvement	19	22) “[Marijuana] also gives me anxiety because I have a friend who lost her daughter to cps for a couple years purely from smoking weed.” (ID #1218) 23) “I was investigated by cps because my hospital turned me in for marijuana in my baby’s cord blood even though my doctor and midwife okayed it because I had [hyperemesis gravidarum]. CPS stayed in my life for 30 days and closed the case with no charges.” (ID # 1231) 24) “I quit smoking around 30 weeks pregnant so my babies would not test positive if drug tested at birth, then resumed smoking as soon as we returned home from the hospital.” (ID # 2164)

^a^Responses could be categorized under more than one theme or subtheme.

#### Theme 1: Cannabis preferred over medications

Nearly half (*n* = 195) of respondents to the open-ended question described using cannabis as a treatment for medical conditions (Table 4). The most mentioned symptoms/conditions were nausea/vomiting, anxiety, depression, pain, and low appetite. History of trauma, insomnia, attention-deficit/hyperactivity disorder (ADHD), Tourette’s syndrome, and inflammatory conditions were also mentioned. Many mothers described using cannabis to address more than one problem (ID #118, Table 4).

**Table 4.** Medical Conditions for Which Cannabis Was Used as a Treatment as Reported in Open-Text Responses (*n* = 195) and Example Quotations

Medical condition	No. of responses^a^	Example quotations
Nausea/Vomiting	81	25) “I had more negative side effects with the medication they gave me for nausea, sleep etc. I chose cannabis because I did not have those side effects...I would do it again if I had any more children.” (ID #1180) 26) “I continued my cannabis use though my doctors tried to push Zofran on me for my daily nausea, I chose cannabis because I was educated in a state funded program about medical cannabis use and decided the risks of a plant were less than the risks of big pharma’s chemical concoction.” (ID #265)
Anxiety	64	27) “On a daily basis, I live with crippling anxiety that renders me useless. I use cannabis to treat my anxiety. When my anxiety is regulated, I am able to be the best mother possible for my children.” (ID #67) 28) “I suffer from severe clinical anxiety and when I got pregnant with my second son my anxiety only increased…Then I started to use cannabis during my pregnancy and my symptoms almost immediately subsided. I felt free and energetic and overall happier throughout my pregnancy…My doctor had wanted to put me back on the anxiety medication…But after seeing improvement, she rescinded the recommendation as long as I was continuing to improve.” (ID #1086)
Depression	48	29) “I abstained during pregnancy until I became suicidally depressed, at which point I found that cannabis helped me not want to kill myself but also helped me sleep despite the heartburn, nightmares, and restless leg syndrome and helped me eat more than cream of wheat and saltines.” (ID #469) 30) “Medical marijuana, in addition to cognitive behavioral therapy, has helped me go from someone who couldn’t function from anxiety/depression to someone who could.” (ID #564)
Pain	35	31) “[After my baby was born] I smoked to deal with surgery recovery pain and stress.” (ID #602) 32) “I use cannabis as an alternative to sedating medications for my severe chronic pain, after a thorough risk/benefit analysis comparing my treatment options. I have found that my pain and quality of life are drastically improved by cannabis. I use primarily CBD.” (ID #860) 33) “I began to use cannabis in place of opiate pain medication for a back injury sustained during my youngest child’s delivery…I could barely walk, was in excruciating pain and had zero quality of life…It has been a lifesaver and safe for my family.” (ID #870)
Increased appetite	33	34) “I mainly used cannabis during my pregnancies because I had been a user and had frequent nausea and vomiting the entire length of pregnancy. Cannabis is the only thing that kept me eating during my first pregnancy.” (ID #1210) 35) “Cannabis made my nausea go away completely and stimulated my appetite.” (ID #1788) 36) “I have a drunk father who left me with a lot of issues when he abandoned my family after I turned 18…I still deal with trauma from him. I can’t afford therapy, but I can afford cannabis and it helps a lot.” (ID #368) 37) “Trauma makes you react in a way that isn’t ‘you’ but rather a memory of what you’ve seen, and marijuana allows me to always be myself and have control over my actions. It’s a level of control I have never thought I could have and I’m so glad I can be my best self with my children.” (ID #898)
Insomnia	18	38) “I used MJ with both my pregnancies mostly in the beginning due to severe N&V. Then used it during breastfeeding both children due to sleep issues/insomnia.” (ID #1169) 39) “I have used CBD tinctures to help with anxiety and insomnia, because I was more comfortable with these than ambien and Zoloft while BF.” (ID #511)
Unspecified condition	16	40) “I had to wean off all of my other prescription medicines when I was pregnant. I spoke to other mothers who had smoked during pregnancy and breastfeeding. I also looked up what research I could.” (ID #1233) 41) “Weed is great I use it over prescription drugs. I also have 4 kids who I all smoked during pregnancy with and they are above average and extremely smart for their age!” (ID #1263) 42) “Medical marijuana can be beneficial if not abused.” (ID#1699)
ADHD	4	43) “I was diagnosed with ADHD at 16 and am currently unmedicated due to the cost of prescription. Mainly, I use to be able to slow down my thoughts and focus on one thing at a time such as completing household tasks.” (ID #57) 44) “For my 4th pregnancy I started using medical cannabis. I had been diagnosed with depression, PTSD, anxiety, ADHD, ODD, mood disorders; reason why my doctor recommended the use of medical cannabis.” (ID #1248)
Autoimmune conditions	3	45) “I am a legalized medical marijuana user for my rheumatoid arthritis, ankylosing spondylitis and Crohn’s Disease… To minimize any potential risks to my son I use a high CBD, low THC marijuana.” (ID #330) 46) “I suffer from severe rheumatoid arthritis, I could easily take opioids for the pain but they’re useless and dangerous. Marijuana essentially gave me my life back, it’s the only way I’m able to function.” (ID #570)

^a^ Responses could be categorized under more than one medical condition if more than one was described in the response.

ADHD, attention-deficit/hyperactivity disorder; PTSD, post-traumatic stress disorder.

Among those describing cannabis use for medical conditions, it was often described as necessary for the survival of themselves or their babies (ID #1505, Table 3). Use of emotional language by the mothers demonstrated how strongly they felt about perinatal cannabis use as helpful to them.

Cannabis was perceived to have fewer or no side effects compared with medications. Specific side effects being avoided were rarely mentioned and medications were typically referred to by class (ID #2051, Table 3). In some cases, participants used cannabis to avoid medication side effects in the infants (ID #2051, Table 3).

Cannabis was described as a “more natural” or “safer” option. It was referred to as an “ancestral herb,” a “natural chemical,” and a “lifesaving medicine.” Because it was perceived as natural, mothers shared that using it did not concern or worry them (ID #933, Table 3).

A few described shared decision-making to use cannabis perinatally. They reported involving other mothers who had used cannabis during their pregnancy, family, and health care professionals. One mother described talking with her doctor about cannabis use for morning sickness (ID #1109, Table 3).

#### Theme 2: Positive impact of cannabis on parent and child

Cannabis use improved quality of life. Mothers felt it helped them be a “better parent” by making them “calmer” or “more present and attentive.” Some mothers explicitly tied being a better parent to using cannabis, which successfully addressed symptoms like anxiety and panic attacks (ID #1105 and #1841, Table 3). For others, this was less explicit (ID #1939, Table 3).

Mothers perceived positive effects on their infants. Many described their children meeting or exceeding milestones despite “birth weight and cognitive development” warnings during their pregnancy. Some reported their children were exceeding milestones and “advanced for their age.” Others noted their child being more expressive and happier (ID #1772, Table 3).

#### Theme 3: Concerns about negative consequences

Although few negative effects were perceived, some mothers expressed concerns about potential negative consequences of cannabis use and adopted behaviors to avoid them. To avoid exposing infants through breast milk, 26 mothers described timing breastfeeding with cannabis use. The amount of time they waited to breastfeed after using cannabis varied; some reported waiting a few hours, and one waited at least 24 hours. Another mother described using formula rather than breastfeeding after using cannabis (ID #2115, Table 3).

Several described only using cannabis after putting their infants to bed, presumably to increase the length of time between use and interacting with or feeding their infants. Others described avoiding infant smoke exposure by washing hands and changing clothes before picking up their baby (ID #933, Table 3).

Negative consequences described were the possibility of hospital repercussions or involvement of CPS. These consequences were mentioned by 19 mothers. Such fears caused a few women to not reveal cannabis use to their doctors (ID #1415, Table 3). Others described testing positive for cannabis during their delivery, which resulted in anxiety or CPS cases. Another mother ceased cannabis use in her third trimester to avoid a positive test during delivery (ID #2164, Table 3).

## Discussion

The mothers in this study perceived perinatal cannabis use as more natural, more effective, and safer than medicines to address medical conditions. In open-ended comments, respondents described using cannabis for reasons previously reported (anxiety and depression, nausea and vomiting, pain, insomnia, and appetite)^13,20^ and specified use for trauma, ADHD, and autoimmune conditions while pregnant or breastfeeding. The relief from symptoms was great enough some women in our study reported “being saved” by cannabis and described it as working when other medications failed them. The language used by our participants demonstrated strongly held beliefs about the positive health impact of cannabis on mothers and sometimes on their children. In some cases, mothers attributed carrying their pregnancy to term to cannabis, further supporting their beliefs of cannabis being “life-saving.”

Not only was cannabis perceived as more effective at treating medical conditions, it was also perceived to improve overall quality of life and help respondents be better parents. These perceived benefits are consistent with previous research on mothers who used cannabis during pregnancy for parenting-related stress.^15,16,21^ In our study, mothers similarly described how it helped them to be patient and engaged rather than irritable or anxious. A reduction in anxiety and stress is beneficial to both the parent and the child. Postpartum stress, which may include depression, anxiety, and posttraumatic stress disorder, has a negative association with infant outcomes in terms of growth and development, nutrition, and bonding.^22^

A majority (98%) of respondents in this survey perceived cannabis to be a natural alternative to medications. Two-thirds of respondents in our study were “not at all concerned” about cannabis affecting their infant while breastfeeding. In contrast, Odom et al.^18^ reported that only 20% of women perceived no risk of cannabis use while pregnant, and Coy et al.^11^ reported 92% of breastfeeding women felt cannabis use was unsafe during breastfeeding. Differences in these findings may be attributed to our convenience sampling strategy versus the more representative PRAMS and National Survey on Drug Use and Health datasets. Mothers also noticed fewer side effects for them and their children with cannabis compared with medications. The perception that cannabis is a safer option for pregnant and breastfeeding women is consistent with previous literature, particularly for controlling nausea in pregnancy.^21,23^

Few women attributed negative outcomes in their children to their use of cannabis. The lack of perceived negative effects is in contrast with evidence that suggests an association between cannabis use during pregnancy and adverse short-term outcomes (sleep and birthweight) and longer-term effects (attention, hyperactivity, and depression) for the child.^6,24^ The risk of negative outcomes because of cannabis use may be downplayed by mothers who perceive benefits from its use. In addition, clinicians and patients tend to overestimate the risk of dramatic, low-probability outcomes, like large malformations, and underestimate risks of more common outcomes, like low birthweight.^25,26^

Despite perceiving cannabis as a best option and reporting low concern of impacts on their infants, many respondents described behaviors to decrease their infants’ exposure to cannabis through breast milk or smoke. Use of harm-reduction strategies were evident in the open-ended question and the multiple-choice questions in this study. Strategies included washing hands, changing clothes, using cannabis when the child is not present, breastfeeding before using cannabis, and waiting a variable amount of time after cannabis use to breastfeed. The behaviors reported are similar to those recommended to mothers who smoke cigarettes or drink alcohol while breastfeeding. Previous studies have reported behaviors for reducing secondhand smoke exposure include smoking in a designated room away from a child to reduce environmental exposure.^27^ Current recommendations on alcohol consumption during breastfeeding advise waiting 90–120 minutes after drinking a serving of alcohol to breastfeed or expressing and discarding the breast milk from that timeframe.^8^ There is currently no standard recommended time to wait after using cannabis to breastfeed.^4^

Nearly half of respondents altered breastfeeding habits to reduce cannabis exposure to the infant. Cannabis has been detected in breast milk from 4 hours up to 6 weeks after its use. Factors that affect the THC content of breast milk include frequency of use, time since most recent use, and THC concentration of the cannabis, which is currently much higher than 20 years ago.^3,28^ Although there is no consensus on how to adjust breastfeeding for cannabis use, there is agreement on advising mothers who consume cannabis through smoking to take measures to reduce second-hand smoke exposure to reduce the risk of sudden infant death syndrome.^29,30^

Fear of CPS involvement or other legal or social repercussions led some mothers to reduce their cannabis use or adopt strategies as mentioned earlier to reduce their child’s exposure. This fear was also a reason for withholding information about cannabis use from their health care provider. CPS involvement and social repercussions have been previously reported as motivators for reducing or discontinuing cannabis use during pregnancy.^31^ The counseling approach of clinicians may often rely on the legal aspect of use rather than the potential health effects of cannabis use during pregnancy and breastfeeding.^32^ Clinical guidelines from ACOG recommend discouraging pregnant and breastfeeding women from using cannabis, and counseling patients that the purpose of cannabis screening is to treat substance use not to punish or prosecute them.^9^ However, it is also recommended to inform the mother of any potential ramifications of positive test results.^9^

Interestingly, using cannabis during pregnancy or while breastfeeding was described by some as a shared decision; partners, friends, family, and even health care providers engaged in the decision to use cannabis perinatally. There is little known about the influence of health care providers, partners, and family on the decision to use cannabis in the postpartum period. A 2017 study reported that mothers who quit smoking cannabis during their pregnancy were more likely to be motivated by the belief that it could harm their pregnancy while few decreased or ceased cannabis use simply because they were advised by their doctor to do so.^16,31^ Furthermore, including warning signs against use of cannabis on cannabis-containing products during pregnancy was not associated with beliefs about its safety among those who used it.^33^ However, it increased support for punishment and stigma among those who did not use cannabis.^33^

## Strengths and Limitations

This study had a large sample size of women who used cannabis while breastfeeding, whereas previous studies have focused on cannabis use during pregnancy.^16^ Coy et al. also examined perceptions of risk associated with cannabis use in the postpartum period but was limited by closed-ended survey questions. The large number of open-text responses in this study provides insight into how or why benefits and risks of cannabis use are perceived and managed among women who use cannabis while breastfeeding. Lack of substantial differences between open-ended question respondents and nonrespondents improves our confidence that the qualitative findings may not be solely unique to this subset.

This research has several limitations. First, our sampling strategies limit the representativeness of all breastfeeding women who have used cannabis, and thus, the generalizability of our findings. The survey was distributed in one support group of mothers who used cannabis, and thus, frequent cannabis users may be overrepresented in this sample possibly skewing in favor of more positive perceptions. Second, the low representation of minorities in this study may be in part because of differences in rates of cannabis use or racial disparity in breastfeeding.^13,34^ This study had a large portion of White, non-Hispanic respondents. This group also has higher rates of both cannabis use and breastfeeding initiation and continuation than black women, which may have contributed to our low sample of individuals who identified as black or people of color.^34^ When informed of studies and given equal access, minorities in the United States have shown similar willingness to participate in health research.^35^ However, it is possible that our sampling strategy did not provide equal access to minorities given the survey’s distribution through a specific website, social media outlets, and snowball sampling.

Another limitation of this study was the inability to further explore perceptions and behaviors described in the open-ended responses because of the nature of the data collection method. For example, the exact medical conditions for which a respondent used cannabis were not always stated, or some of the responses provided were vague or unclear.

## Conclusions

As cannabis legalization continues, its use among breastfeeding women may also continue to rise. This study provides insights into decision-making among breastfeeding women who use cannabis and their strategies to reduce risks for their infants. Breastfeeding women who used cannabis perceived it as safer than medications; some perceived benefits to cannabis. It was perceived to reduce stress and allow them to be more attentive to their children and engaged as parents. Few mothers who used cannabis perceived negative outcomes; however, nearly half of respondents reported harm-reduction strategies. Several participants reported concern about the risk of legal repercussions. Focusing counseling on legal and social repercussions may discourage some mothers from sharing information with their providers. Practitioners should hold open nonjudgmental conversations about cannabis use in the perinatal period and counsel on risk–benefit analysis while being aware of parents’ concerns of possible CPS involvement. Future studies should examine patient counseling, the decision-making process around perinatal cannabis use, and further explore health outcomes in the parent and child with cannabis use. This would give health care providers the information necessary to be prepared participants in the shared decision-making process.

## Abbreviations Used

ACOG: American College of Obstetricians and Gynecologists
ADHD: Attention-Deficit/ Hyperactive Disorder
BIPOC: Black, Indigenous, or Other People of Color
CPS: Child Protective Services
IRB: Institutional Review Board
PRAMS: Pregnancy Risk Assessment Monitoring System
PTSD: Post-Traumatic Stress Disorder
THC: D9-tetrahydrocannabinol

## References

[1] State Medical Cannabis Laws. 2023. Available from: https://www.ncsl.org/health/state-medical-cannabis-laws

[2] Skelton KR, Hecht AA, Benjamin-Neelon SE. Recreational cannabis legalization in the US and maternal use during the preconception, prenatal, and postpartum periods. Int J Environ Res Public Health 2020;17(3):909. 32024173 10.3390/ijerph17030909 PMC7037220

[3] ElSohly MA, Chandra S, Radwan M, et al. A comprehensive review of cannabis potency in the United States in the last decade. Biol Psychiatry: Cogn Neurosci Neuroimaging 2021;6(6):603–606. 33508497 10.1016/j.bpsc.2020.12.016

[4] Baker T, Datta P, Rewers-Felkins K, et al. Transfer of inhaled cannabis into human breast milk. Obstet Gynecol 2018;131(5):783–788; doi: 10.1097/AOG.0000000000002575 29630019

[5] Moss MJ, Bushlin I, Kazmierczak S, et al. Cannabis use and measurement of cannabinoids in plasma and breast milk of breastfeeding mothers. Pediatr Res 2021;90(4):861–868; doi: 10.1038/s41390-020-01332-2 33469174

[6] Metz TD, Borgelt LM. Marijuana use in pregnancy and while breastfeeding. Obstet Gynecol 2018;132(5):1198–1210; doi: 10.1097/Aog.0000000000002878 30234728 PMC6370295

[7] Goldschmidt L, Richardson GA, Willford J, et al. Prenatal marijuana exposure and intelligence test performance at age 6. J Am Acad Child Adolesc Psychiatry 2008;47(3):254–263; doi: 10.1097/CHI.0b013e318160b3f0 18216735

[8] Reece-Stremtan S, Marinelli KA. ABM clinical protocol #21: Guidelines for breastfeeding and substance use or substance use disorder, revised 2015. Breastfeed Med 2015;10(3):135–141; doi: 10.1089/bfm.2015.9992 25836677 PMC4378642

[9] Committee opinion no. 722: Marijuana use during pregnancy and lactation. Obstet Gynecol 2017;130(4):e205–e209; doi: 10.1097/aog.0000000000002354 28937574

[10] Crume TL, Juhl AL, Brooks-Russell A, et al. Cannabis use during the perinatal period in a state with legalized recreational and medical marijuana: The association between maternal characteristics, breastfeeding patterns, and neonatal outcomes. J Pediatr 2018;197:90–96. 29605394 10.1016/j.jpeds.2018.02.005

[11] Coy KC, Haight SC, Anstey E, et al. Postpartum marijuana use, perceptions of safety, and breastfeeding initiation and duration: An analysis of PRAMS data from seven states, 2017. J Hum Lact 2021;37(4):803–812; doi: 10.1177/0890334421993466 33586506 PMC8361861

[12] Garner CD, Kendall-Tackett K, Young C, et al. Mode of cannabis use and factors related to frequency of cannabis use among breastfeeding mothers: Results from an online survey. Breastfeed Med 2022;17(3):269–276; doi: 10.1089/bfm.2021.0151 34870449

[13] Ko JY, Coy KC, Haight SC, et al. Characteristics of marijuana use during pregnancy—eight states, pregnancy risk assessment monitoring system, 2017. MMWR Morb Mortal Wkly Rep 2020;69(32):1058–1063; doi: 10.15585/mmwr.mm6932a2 32790656 PMC7440118

[14] Young-Wolff KC, Gali K, Sarovar V, et al. Women's questions about perinatal cannabis use and health care providers' responses. J Womens Health (Larchmt) 2020;29(7):919–926. 32011205 10.1089/jwh.2019.8112 PMC7371546

[15] Dreher MC. Poor and pregnant: Perinatal ganja use in rural Jamaica. Adv Alcohol Subst Abuse 1989;8(1):45–54. 2785329 10.1300/J251v08n01_03

[16] Vanstone M, Panday J, Popoola A, et al. Pregnant people's perspectives on cannabis use during pregnancy: A systematic review and integrative mixed-methods research synthesis. J Midwifery Womens Health 2022;67(3):354–372; doi: 10.1111/jmwh.13363 35445514 PMC9324983

[17] Oh S, Salas-Wright CP, Vaughn MG, et al. Marijuana use during pregnancy: A comparison of trends and correlates among married and unmarried pregnant women. Drug Alcohol Depend 2017;181:229–233. 29107787 10.1016/j.drugalcdep.2017.09.036

[18] Odom GC, Cottler LB, Striley CW, et al. Perceived risk of weekly cannabis use, past 30-day cannabis use, and frequency of cannabis use among pregnant women in the United States. Int J Womens Health 2020;12:1075–1088. 33235517 10.2147/IJWH.S266540 PMC7678496

[19] Regalado D, Connolly ME, Krutsch K, et al. Psychiatric medication use among pregnant and breastfeeding mothers who used cannabis for mental health concerns: A cross-sectional survey study. Womens Health (Lond) 2023;19:17455057231199391; doi: 10.1177/17455057231199391 37746858 PMC10521288

[20] Swenson K. Cannabis for morning sickness: Areas for intervention to decrease cannabis consumption during pregnancy. J Cannabis Res 2023;5(1):22; doi: 10.1186/s42238-023-00184-x 37330589 PMC10276456

[21] Barbosa-Leiker C, Burduli E, Smith CL, et al. Daily cannabis use during pregnancy and postpartum in a state with legalized recreational cannabis. J Addict Med 2020;14(6):467–474; doi: 10.1097/adm.0000000000000625 32011411 PMC7647431

[22] Oyetunji A, Chandra P. Postpartum stress and infant outcome: A review of current literature. Psychiatry Res 2020;284:112769; doi: 10.1016/j.psychres.2020.112769 31962260

[23] Chang JC, Tarr JA, Holland CL, et al. Beliefs and attitudes regarding prenatal marijuana use: Perspectives of pregnant women who report use. Drug Alcohol Depend 2019;196:14–20. 30658220 10.1016/j.drugalcdep.2018.11.028 PMC6756431

[24] Paul SE, Hatoum AS, Fine JD, et al. Associations between prenatal cannabis exposure and childhood outcomes: Results from the ABCD study. JAMA Psychiatry 2021;78(1):64–76; doi: 10.1001/jamapsychiatry.2020.2902 32965490 PMC7512132

[25] Thürmann PA. Safety and risk communication to patients. Expert Opin Drug Saf 2006;5(6):747–750; doi: 10.1517/14740338.5.6.747 17044801

[26] Widnes SF, Schjøtt J. Risk perception regarding drug use in pregnancy. Am J Obstet Gynecol 2017;216(4):375–378; doi: 10.1016/j.ajog.2016.12.007 27988271

[27] Cartmell KB, Miner C, Carpenter MJ, et al. Secondhand smoke exposure in young people and parental rules against smoking at home and in the car. Public Health Rep 2011;126(4):575–582; doi: 10.1177/003335491112600414 21800752 PMC3115217

[28] Volkow ND, Baler RD, Compton WM, et al. Adverse health effects of marijuana use. N Engl J Med 2014;370(23):2219–2227. 24897085 10.1056/NEJMra1402309 PMC4827335

[29] Meek JY, Noble L. Technical report: Breastfeeding and the use of human milk. Pediatrics 2022;150(1); doi: 10.1542/peds.2022-057989 35921641

[30] Klonoff-Cohen H, Lam-Kruglick P. Maternal and paternal recreational drug use and sudden infant death syndrome. Arch Pediatr Adolesc Med 2001;155(7):765–770; doi: 10.1001/archpedi.155.7.765 11434841

[31] Mark K, Gryczynski J, Axenfeld E, et al. Pregnant women's current and intended cannabis use in relation to their views toward legalization and knowledge of potential harm. J Addict Med 2017;11(3):211–216; doi: 10.1097/adm.0000000000000299 28252456

[32] Panday J, Taneja S, Popoola A, et al. Clinician responses to cannabis use during pregnancy and lactation: A systematic review and integrative mixed-methods research synthesis. Fam Pract 2022;39(3):504–514. 34791187 10.1093/fampra/cmab146 PMC9155166

[33] Roberts SC, Zaugg C, Biggs MA. Association of mandatory warning signs for cannabis use during pregnancy with cannabis use beliefs and behaviors. JAMA Netw Open 2023;6(6):e2317138–e2317138. 37314807 10.1001/jamanetworkopen.2023.17138 PMC10267765

[34] Jones KM, Power ML, Queenan JT, et al. Racial and ethnic disparities in breastfeeding. Breastfeed Med 2015;10(4):186–196; doi: 10.1089/bfm.2014.0152 25831234 PMC4410446

[35] Wendler D, Kington R, Madans J, et al. Are racial and ethnic minorities less willing to participate in health research? PLoS Med 2006;3(2):e19; doi: 10.1371/journal.pmed.0030019 16318411 PMC1298944

